# Good mid‐ to long‐term survivorship and low rates of aseptic loosening of a cementless primary press‐fit acetabular cup for contained defects in revision total hip arthroplasty

**DOI:** 10.1002/jeo2.70871

**Published:** 2026-08-03

**Authors:** Kevin‐Arno Koch, Benjamin Zimmer, Raphael Trefzer, Mustafa Hariri, Tilman Walker, Christian Merle, Johannes Weishorn, David Spranz

**Affiliations:** ^1^ Department of Orthopaedics Heidelberg University Hospital Heidelberg Germany; ^2^ Department of Orthopaedic and Trauma Surgery Paulinenhilfe, Diakonieklinikum Stuttgart Stuttgart Germany

**Keywords:** acetabular cup, cementless fixation, patient reported outcome measures, prosthesis failure, reoperation, revision hip arthroplasty

## Abstract

**Purpose:**

Acetabular revision total hip arthroplasty (rTHA) remains challenging, and evidence on the use of primary press‐fit cups in the revision setting for contained acetabular defects is limited. This study evaluated mid‐ to long‐term implant survivorship, reoperation rates and clinical and radiographic outcomes of a cementless primary press‐fit acetabular cup used for acetabular revision.

**Methods:**

This retrospective single‐centre study included 107 hips in 106 patients undergoing acetabular rTHA using a cementless primary press‐fit cup. Implant survivorship was assessed using cumulative incidence functions for acetabular re‐revision for any reason, re‐revision for aseptic loosening and reoperation for any reason, with death treated as a competing event. Fine and Gray subdistribution hazard models were used to evaluate potential risk factors. Patient‐reported outcome measures included the Harris Hip Score (HHS) and the University of California Los Angeles (UCLA) activity score. Radiographic evaluation was performed using standardised classifications.

**Results:**

Cumulative incidence of acetabular re‐revision for any reason was 5.9% at 5 years and 9.8% at 10 years. Re‐revision for aseptic loosening occurred in two hips (1.9%), corresponding to survivorship of 99.0% at 5 years and 95.0% at 10 years. The cumulative incidence of reoperation for any reason was 21.7% at 10 years. Previous revision surgery was the only factor associated with an increased risk of subsequent failure. Mean HHS increased from 47 to 76, and the median UCLA activity score from 4 to 6. No radiographic acetabular loosening was observed among patients available for radiographic follow‐up.

**Conclusion:**

In selected acetabular revision cases with contained defects, cementless primary press‐fit acetabular cups demonstrated favourable mid‐ to long‐term implant survivorship. Postoperative functional outcomes were comparable to those reported in the existing revision THA literature. Aseptic loosening accounted for only a minority of failures, suggesting durable biological fixation in this revision setting.

**Level of Evidence:**

Level III, retrospective cohort study.

**Abbreviations**
BMI: body mass index
CI: confidence interval
CIF: cumulative incidence function
CT: computed tomography
DAIR: debridement, antibiotics and implant retention
HHS: Harris Hip Score
PJI: periprosthetic joint infection
PROMs: patient‐reported outcome measures
rTHA: revision total hip arthroplasty
SD: standard deviation
sHR: subdistribution hazard ratio
UCLA: University of California Los Angeles Activity Score

## INTRODUCTION

Revision total hip arthroplasty (rTHA) procedures are projected to increase substantially over the coming decades [21, 24]. This trend is particularly relevant in younger and more active patients, who face a higher lifetime risk of revision and often present with preserved acetabular bone stock. Despite advances in implant design and surgical techniques, outcomes following rTHA remain challenging, with an increased risk of re‐revision over time and with each subsequent revision procedure [6]. Appropriate selection of revision strategies and implants is therefore critical to optimise implant survivorship and clinical outcomes.

Failure of the acetabular component remains a leading cause for revision in cementless total hip arthroplasty [9, 30]. Implant selection in acetabular revision surgery is primarily guided by defect morphology and residual bone stock [16, 30]. While revision‐specific acetabular components are commonly used for contained defects, cementless primary press‐fit cups may represent a viable option in selected cases with sufficient circumferential bone support [2, 13, 16]. In addition, primary press‐fit cups are generally associated with lower implant‐related costs compared with dedicated revision systems [8], a relevant consideration given the higher overall resource utilisation in rTHA [18].

Although several studies have reported favourable survivorship of uncemented acetabular components in revision surgery, the available evidence is heterogeneous, with varying implant designs, indications and endpoint definitions [14, 17, 27, 31]. In particular, data on the use of primary press‐fit cups in the revision setting remain limited.

Therefore, the purpose of the present study was to determine the mid‐ to long‐term implant survivorship and revision risk, as well as the clinical and radiographic outcomes, of a cementless primary press‐fit acetabular cup used in acetabular rTHA.

## MATERIALS AND METHODS

### Study design and patient selection

In this single‐centre study, all patients who underwent acetabular revision surgery for contained acetabular defects (Paprosky type I, II and IIIA defects, with >60% peripheral cup coverage) using a cementless primary press‐fit acetabular cup (Allofit or Allofit‐S, Zimmer Biomet) at a tertiary referral centre between September 1999 and December 2011 were consecutively reviewed. The study was conducted in accordance with the Declaration of Helsinki and was approved by the institutional review board. Informed consent was obtained from all participating patients prior to inclusion.

Data were obtained from the institution's database. Acetabular defects were classified according to the Paprosky classification based on preoperative radiographs and intraoperative findings. Computed tomography (CT) imaging was performed selectively in cases where additional assessment of acetabular bone loss was considered necessary for preoperative planning. Patients receiving an acetabular component other than the Allofit/Allofit‐S system or presenting with uncontained acetabular defects (Paprosky type IIIB or IV) were excluded.

The decision to use a primary press‐fit acetabular component in the revision setting was based on preoperative imaging, intraoperative assessment of bone stock and defect morphology, and the ability to achieve stable circumferential press‐fit fixation. This strategy was primarily applied in contained acetabular defects without pelvic discontinuity or the need for extensive augment reconstruction.

Following screening, 106 patients (107 hips) met the inclusion criteria. Baseline patient characteristics are summarised in Table 1 and indications for the index acetabular revision are presented in Table 2.

**Table 1:** Baseline characteristics of the study cohort.

Characteristic	Value
Number of patients/hips	106/107
Age at revision surgery, years (SD, range)	64 (13; 28–86)
Sex	
Male	51 (48%)
Female	56 (52%)
BMI, kg/m^2^ (SD, range)	29.2 (6.1; 20.5–51.4)
Previous revision surgery, *n* (%)	
Yes	10 (9%)
No	97 (91%)

*Note*: Values are presented as mean (SD, range) or *n* (%), as appropriate. Abbreviations: BMI, body mass index; SD, standard deviation.

**Table 2:** Indications for index acetabular revision surgery.

Reason for index acetabular revision	n (%)
Aseptic loosening	69 (64)
Isolated cup loosening	51 (48)
Combined acetabular and femoral loosening	18 (16)
Periprosthetic joint infection	29 (27)
Periprosthetic fracture	4 (4)
Recurrent dislocations	2 (2)
Implant failure	2 (2)
Cup breakage	1 (1)
Liner breakage	1 (1)
Acetabular malposition	1 (1)

### Surgical procedure and implant characteristics

All revision procedures were performed by experienced arthroplasty surgeons in a laminar airflow operating room. One‐stage exchange arthroplasty for aseptic indications was performed in 78 cases, whereas 29 patients underwent two‐stage revision for periprosthetic joint infection.

In all patients, a cementless acetabular component with a bispherical design and polar flattening (Allofit or Allofit‐S, Zimmer Biomet) was implanted, with the latter allowing optional supplementary screw fixation. The implant features a grit‐blasted titanium surface with circumferential macrostructures and equatorial grooves to enhance primary press‐fit and rotational stability. The acetabulum was under‐reamed by 2 mm in the equatorial region in all cases.

Supplementary screw fixation was applied at the discretion of the treating surgeon when enhanced primary stability was considered beneficial, particularly in the presence of reduced bone quality or limited press‐fit fixation. Cancellous bone impaction grafting was performed in contained cavitary defects with localised bone loss when reconstruction of acetabular bone stock was considered necessary to support stable press‐fit fixation. Morselized cancellous graft was manually impacted into the defect bed prior to cup implantation. Radiographic graft incorporation was not systematically evaluated. In 102 cases, a polyethylene liner (Sulene‐PE/Durasul, Zimmer Biomet) was used in combination with either a ceramic head (Biolox option, CeramTec) or a metal head (BioBall, Merete), whereas five patients received a Metasul metal‐on‐metal articulation (Zimmer). The choice of bearing surface was based on implant compatibility, institutional practice during the respective time period, and surgeon preference. No dual‐mobility articulations were used in this cohort. Metal augments were not routinely employed. However, one patient received a trabecular metal wedge for additional acetabular defect reconstruction.

Isolated acetabular revision without femoral stem revision was performed in 54 cases (50%), whereas concomitant femoral reconstruction was required in the remaining 53 hips. Further surgical and implant characteristics are summarised in Table 3.

**Table 3:** Defect classification, surgical characteristics and implant configurations of the study cohort.

Characteristic	Value
Paprosky defect type	
I	66 (62%)
IIA	18 (17%)
IIB	7 (6%)
IIC	6 (6%)
IIIA	10 (9%)
Surgical approach	
Modified transgluteal Bauer	97 (91%)
Modified Watson–Jones	9 (8%)
Posterior	1 (1%)
Mean acetabular cup size, mm (SD, range)	56 (4; 46–68)
Supplementary screw fixation	
Yes	53 (50%)
No	54 (50%)
Bone grafting	
Autologous	11 (10%)
Allogeneic	11 (10%)
None	85 (80%)
Bearing surface	
Ceramic‐on‐polyethylene	56 (52%)
Metal‐on‐polyethylene	46 (43%)
Metal‐on‐metal	5 (5%)
Femoral head sizes, mm	
28	20 (19%)
32	76 (71%)
36	11 (10%)
Femoral reconstruction	
No femoral revision	54 (51%)
Modular tapered uncemented stem	33 (31%)
CLS Spotorno stem	10 (9%)
Other cemented or cementless stems	10 (9%)

*Note*: Values are presented as *n* (%) unless otherwise indicated.

Abbreviations: CLS, CementLess Spotorno stem; SD, standard deviation.

### Clinical and radiographic review

Clinical and radiographic follow‐up examinations were recommended in regular intervals at 3 months, 1 year, 3 years, 5 years and then every 5 years thereafter. Patients who were unable to attend the examination due to health conditions or other reasons were contacted by mail or phone to provide additional information on complications or revision procedures and to complete the patient‐reported outcome measures (PROMs) questionnaires. For deceased patients, information between the last clinical follow‐up and death was obtained using information from relatives, general practitioners and hospital records. Patients who underwent re‐revision and those who died were not included in subsequent clinical and radiographic outcome analyses.

For the present study, PROMs were evaluated at the most recent follow‐up. The Harris Hip Score (HHS) and University of California Los Angeles (UCLA) activity score were recorded.

For radiographic evaluation, patients underwent standardised anteroposterior pelvic radiographs and lateral (Lauenstein) radiographs of the hip. Periacetabular radiolucency and osteolysis were graded according to the three zones as described by DeLee and Charnley [7]. Osteolysis was defined as a progressive radiolucent area in the bone‐prosthesis interface on serial radiographs [32]. Acetabular loosening was defined as a horizontal or vertical implant migration of more than 2 mm, a change in inclination or version of >5° or a complete radiolucent line in all DeLee and Charnley zones. Heterotopic ossification was graded according to Brooker et al. [3].

Radiographic evaluations were performed independently by two investigators, including one fellowship‐trained orthopaedic surgeon. Formal interobserver reliability analysis was not performed.

### Statistical analysis

Data were analysed using SPSS Statistics Version 31.0 and R Version 4.5.2. Statistical significance was defined as *p* < 0.05. Continuous variables are reported as means with standard deviations, except for the UCLA score, which is presented as median and range. Normality was assessed using the Shapiro–Wilk test.

Implant survival was evaluated using cumulative incidence functions (CIFs) for three endpoints: acetabular re‐revision for any reason, re‐revision for aseptic loosening and reoperation for any reason, with death treated as a competing event. Reoperation was defined as any subsequent surgical procedure related to the revised hip joint, irrespective of implant exchange or component retention, including debridement, liner exchange, open reduction or repeat revision surgery. Time‐to‐event analyses were performed according to Fine and Gray subdistribution hazard model. Patients were censored at last follow‐up or at procedures not meeting the respective endpoint definition. Ten‐year survival estimates were generated, with 16 hips remaining at risk for acetabular re‐revision and for aseptic loosening, and 12 hips at risk for reoperation at the final follow‐up interval. Ninety‐five per cent confidence intervals were derived from CIF variance estimates.

Associations with potential risk factors were explored using Fine and Gray subdistribution hazard models. Univariate analyses included age, BMI, sex, previous revision surgery (defined as any revision procedure performed between the primary implantation and the index acetabular revision), septic versus aseptic indication and use of supplementary screws. Owing to the limited number of events, multivariable modelling was restricted to the reoperation endpoint and included age and previous revision status.

## RESULTS

### Study cohort

From the initial cohort (107 hips in 106 patients), 5 patients (5 hips, 4.6%) were lost to follow‐up. During the study period, 19 patients (19 hips, 17.8%) had died from unrelated causes with the implant in situ. At final follow‐up, 7 patients (7 hips, 6.5%) had undergone re‐revision of the acetabular component, with two patients due to aseptic loosening (2 hips, 1.9%). A total of 20 patients had undergone reoperations (20 hips, 18.7%). The mean follow‐up duration for the overall cohort was 7.2 years (SD 4.1; range, 1–19 years).

**Figure 1:**
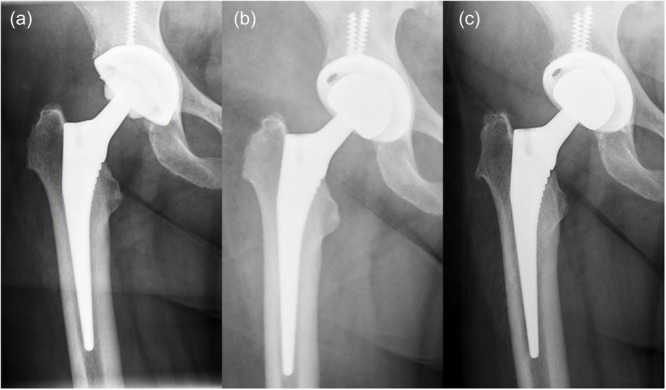
Illustrative radiographs of a 59‐year‐old female patient undergoing acetabular re‐revision total hip arthroplasty for aseptic loosening of a previously revised acetabular component. An Allofit‐S cementless primary press‐fit acetabular cup with two supplementary screws was implanted. (a) Preoperative. (b) Immediate postoperative. (c) Thirteen‐year follow‐up demonstrating stable implant fixation without radiographic evidence of loosening.

**Figure 2:**
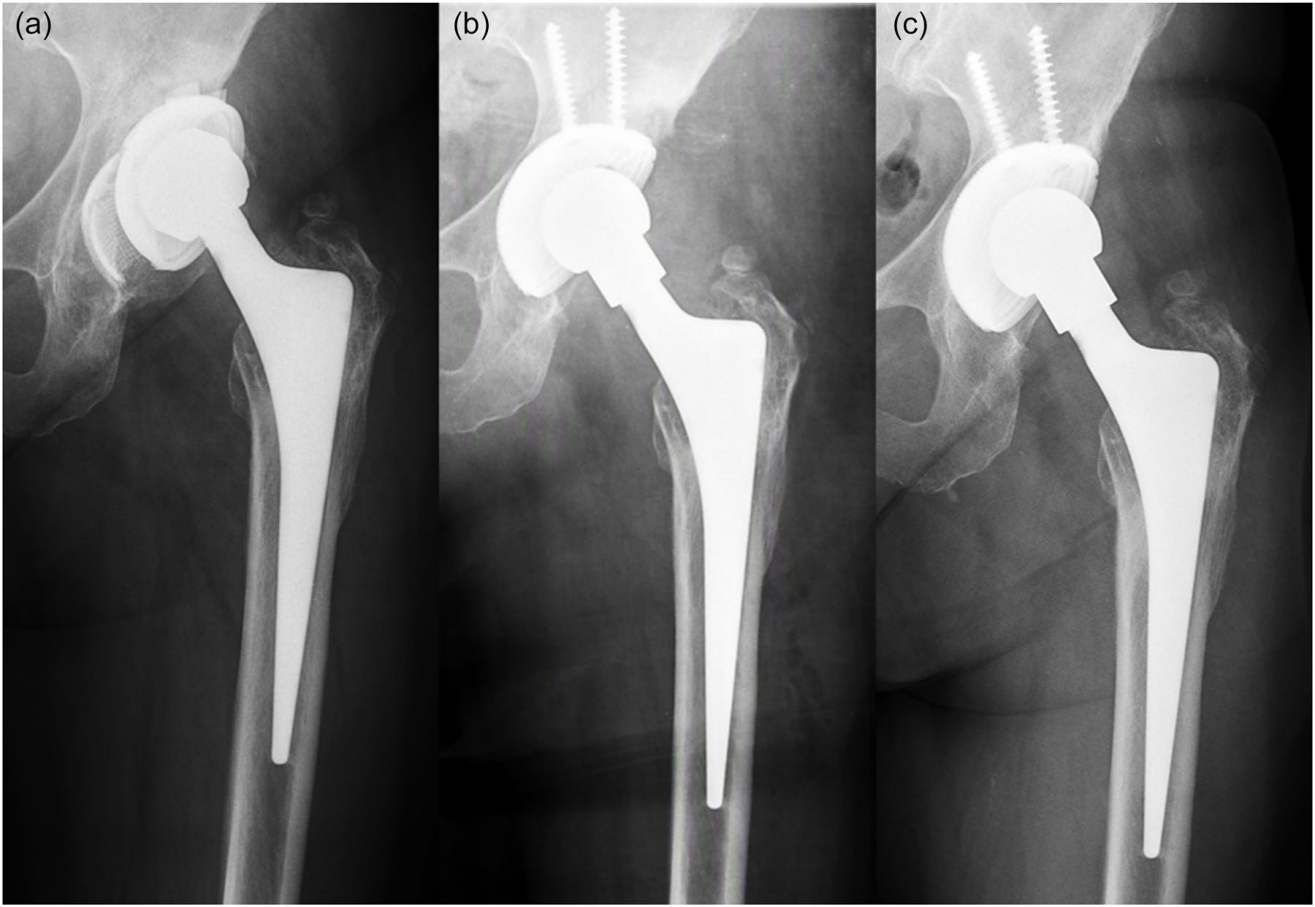
Illustrative radiographs of a 55‐year‐old female patient undergoing acetabular revision total hip arthroplasty for aseptic loosening secondary to implant failure. An Allofit‐S cementless primary press‐fit acetabular cup with two supplementary screws was implanted. (a) Preoperative. (b) Immediate postoperative. (c) Five‐year follow‐up demonstrating stable implant fixation without radiographic evidence of loosening.

Representative radiographic examples demonstrating mid‐ to long‐term implant stability following acetabular revision using a cementless primary press‐fit cup are shown in Figures 1 and 2.

### Re‐revisions and reoperations

In total, 7 patients underwent acetabular re‐revision surgery, including revision of the acetabular cup. The mean time to re‐revision was 30 months (range, 0–119 months). The details of re‐revision surgery are provided in Table 4.

**Table 4:** Details of acetabular re‐revisions during follow‐up.

Case	Time to re‐revision, months	Reason for index revision	Index procedure	Initial paprosky defect	Supplementary screw fixation	Reason for re‐revision
1	1	Aseptic loosening	Isolated cup revision	IIIA	Yes	Recurrent instability
2	1	Aseptic loosening	Isolated cup revision	I	No	Acute PJI
3	1	Aseptic loosening	Isolated cup revision	I	No	Acute PJI
4	4	PJI	Two‐stage revision	I	Yes	Acute PJI
5	39	PJI	Two‐stage revision	IIA	No	Chronic PJI
6	47	Aseptic loosening	Isolated cup revision	I	Yes	Aseptic loosening
7	119	Aseptic loosening	Isolated cup revision	IIA	No	Aseptic loosening

Abbreviation: PJI, periprosthetic joint infection.

In addition, 13 further re‐reoperations were performed, comprising 7 isolated femoral stem revisions (5 aseptic loosenings, 1 periprosthetic fracture and 1 breakage of the stem), 2 DAIR (debridement, antibiotics and implant retention) procedures for acute PJI, 2 revisions for recurrent dislocation (1 isolated inlay and femoral head exchange, 1 inlay and modular femoral neck revision), 1 isolated inlay revision due to adverse reaction to metal debris related to a metal‐on‐metal bearing and 1 haematoma evacuation.

### Survival analysis

**Figure 3:**
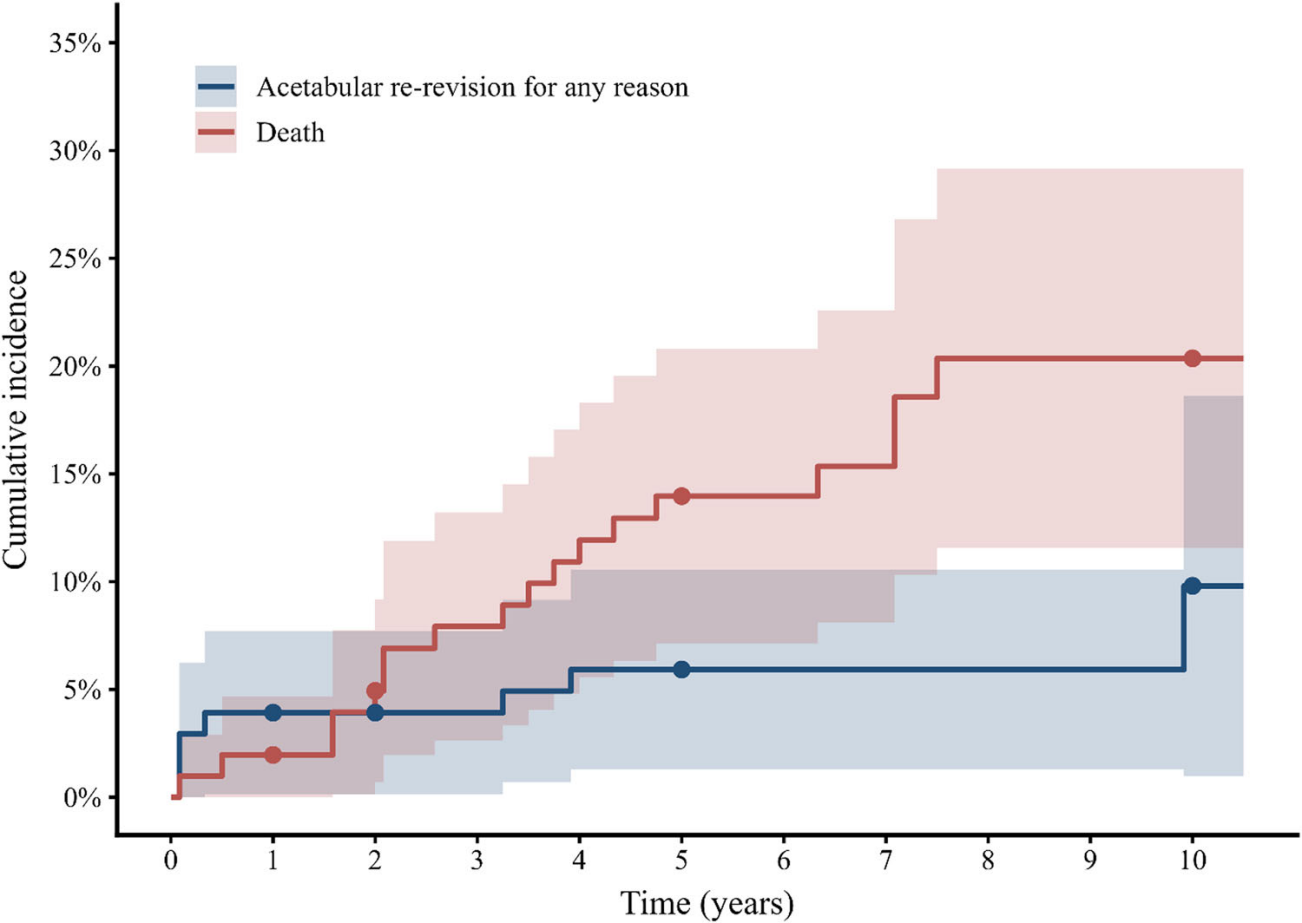
Cumulative incidence of acetabular re‐revision for any reason with death as a competing risk.

The cumulative incidence of acetabular re‐revision for any reason was 3.9% (95% CI: 0.1%–7.7%) at both 1 and 2 years, 5.9% (1.3%–10.5%) at 5 years and 9.8% (1.0%–18.6%) at 10 years. The competing risk of death was 2.0% (95% CI: 0.0%–4.7%) at 1 year, 4.9% (0.7%–9.2%) at 2 years, 14.0% (7.1%–20.8%) at 5 years and 20.4% (11.6%–29.2%) at 10 years (Figure 3).

**Figure 4:**
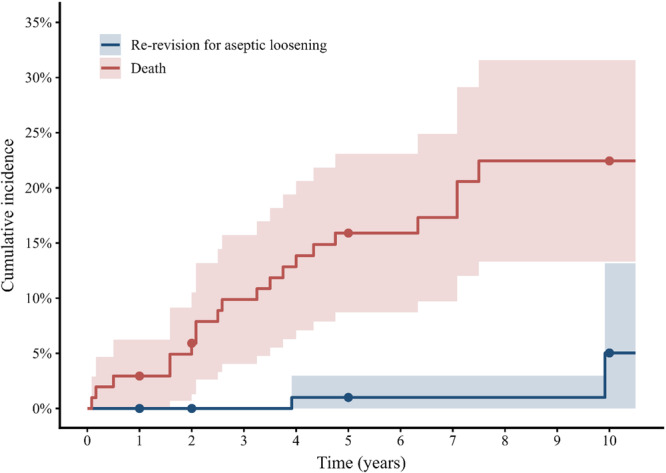
Cumulative incidence of re‐revision for aseptic loosening with death as a competing risk.

For re‐revision for aseptic loosening, the cumulative incidence remained 0.0% (95% CI: 0.0%–0.0%) at 1 and 2 years, 1.0% (0.0%–3.0%) at 5 years and 5.0% (0.0%–13.2%) at 10 years. The competing risk of death was 2.9% (95% CI: 0.0%–6.2%) at 1 year, 5.9% (1.3%–10.5%) at 2 years, 15.9% (8.7%–23.1%) at 5 years and 22.4% (13.3%–31.6%) at 10 years (Figure 4).

**Figure 5:**
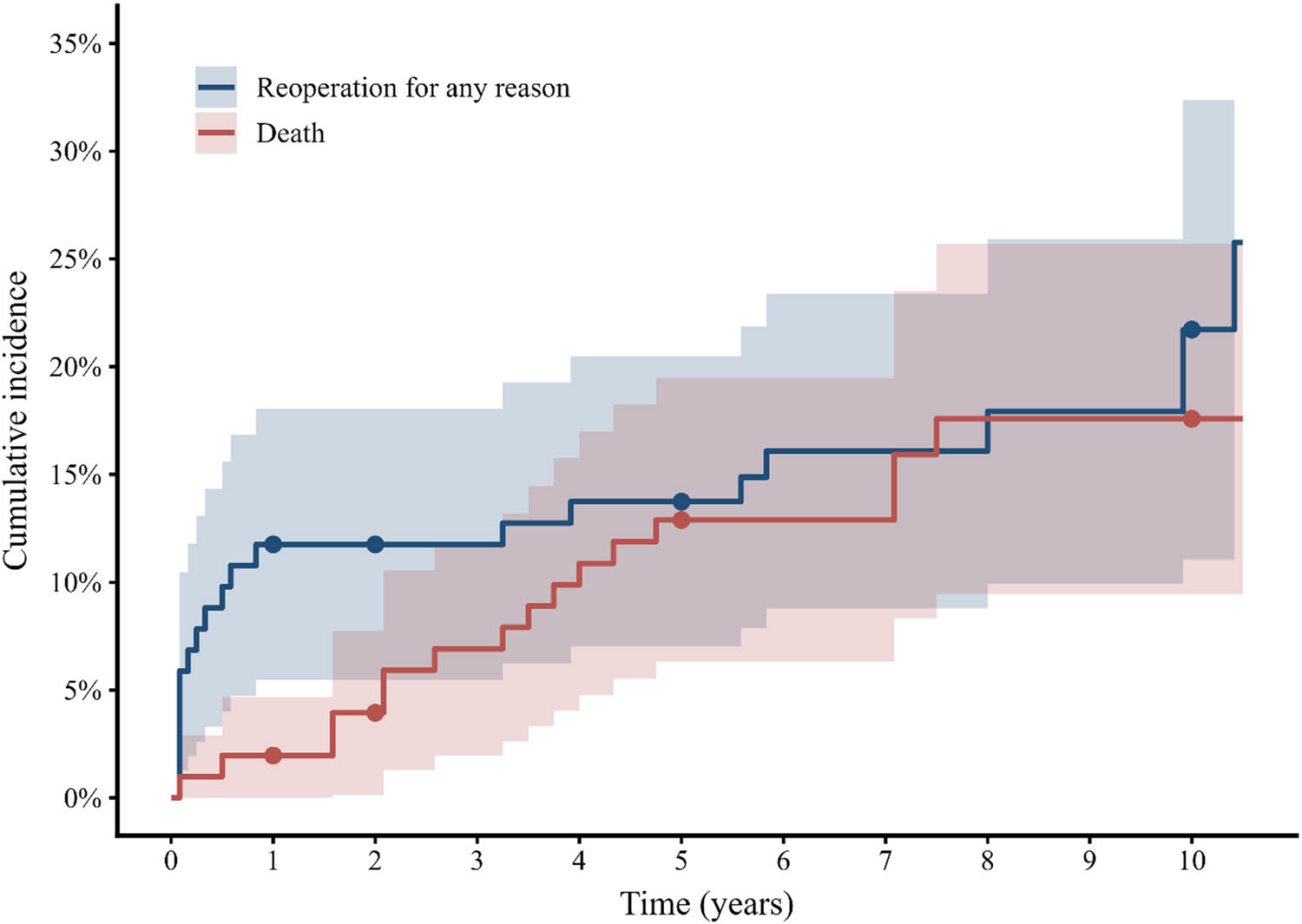
Cumulative incidence of reoperation for any reason with death as a competing risk.

For reoperation for any reason, the cumulative incidence was 11.8% (95% CI: 5.5%–18.0%) at 1 and 2 years, 13.7% (7.0%–20.5%) at 5 years and 21.7% (11.1%–32.4%) at 10 years. The competing risk of death occurred in 2.0% (95% CI: 0.0%–4.7%) at 1 year, 3.9% (0.1%–7.8%) at 2 years, 12.9% (6.3–19.5%) at 5 years and 17.6% (9.5%–25.7%) at 10 years (Figure 5).

### Risk factor analysis

For the endpoint acetabular re‐revision for any reason, univariate Fine and Gray analyses demonstrated no significant association with age, BMI, sex, septic indication or use of supplementary screws (*p* > 0.05). Previous revision was the only factor associated with an increased risk (subdistribution hazard ratio [sHR]: 10.1; 95% CI: 2.1–47.9).

For the endpoint reoperation for any reason, univariate analyses showed no significant effect of age, BMI, sex, septic indication or use of supplementary screws. Previous revision remained the sole factor associated with higher risk (sHR: 3.2; 95% CI: 1.1–9.8). In the multivariable model including age and previous revision, age did not materially influence risk, whereas previous revision continued to be associated with increased hazard (sHR: 3.5; 95% CI: 1.2–10.6).

### Functional outcome

PROMs were available for 72 patients at the most recent follow‐up, with a mean follow‐up of 8 years (range, 1–19 years). The mean HHS increased from 47 preoperatively (SD: 23.2; range, 10–98) to 76 (SD: 23; range, 15–100) at latest follow‐up. The UCLA Activity Score increased from a median of 4 preoperatively (range, 1–10) to 6 (range, 2–9) at final follow‐up.

### Radiographic evaluation

Radiographic follow‐up was available for 60 patients. None showed radiographic signs of acetabular component loosening. One patient demonstrated small periacetabular osteolytic lesions (<2 mm) located in DeLee–Charnley zones I and III. Another patient exhibited pronounced polyethylene wear 12 years after implantation, without associated radiolucent lines around the cup. Heterotopic ossification was identified in 13 patients. According to the Brooker classification, 5 patients were graded as Brooker I (one symptomatic), 4 as Brooker II (one symptomatic), 3 as Brooker III (one symptomatic) and 1 as Brooker IV (one symptomatic).

## DISCUSSION

The most important finding of this study was that a cementless primary acetabular press‐fit cup achieved good mid‐ to long‐term implant survivorship in acetabular revision arthroplasty, with cumulative failure rates of 5.9% at 5 years and 9.8% at 10 years. Although the overall reoperation rate was substantial, aseptic loosening and cup re‐revision were scarce, indicating durable biological fixation in appropriately selected cases. Previous revision surgery emerged as the only identifiable risk factor for subsequent failure, highlighting the clinical relevance of revision burden.

Acetabular revision surgery remains challenging, requiring individualised implant selection based on bone stock and defect morphology. Recent technological developments, including robotic‐assisted revision arthroplasty and emerging applications of artificial intelligence, may further support preoperative planning and decision‐making in complex hip reconstruction [15, 29].

While porous‐coated revision cups with augments are preferred in cases with uncontained defects or pelvic discontinuity and structural bone grafting may be required for reconstruction of massive acetabular defects [1, 5, 25], contained defects can be reliably addressed using cementless press‐fit components when adequate circumferential fixation is achievable [13, 20, 22].

Published outcomes for uncemented acetabular revision components consistently demonstrate favourable mid‐ to long‐term survivorship [10, 12, 14, 17, 30, 31]. Studies of porous‐coated cups have reported implant survival rates ranging from approximately 89% to over 97% across varying endpoints and follow‐up durations, including re‐revision for any reason and aseptic loosening [14, 17, 31]. A large registry analysis further supports these findings, showing survivorship ≥95% at 2–10 years for contemporary revision‐specific designs [30]. However, heterogeneity in implant types, surgical indications and definitions of failure complicates direct comparison between studies.

The present study adds to this literature through evaluation of the Allofit cup, a cementless, grit‐blasted, non–porous‐coated primary press‐fit implant used in a revision setting. To our knowledge, no previous study has specifically reported mid‐ to long‐term outcomes for this implant in acetabular revision surgery. The Allofit design with a thin shell and macrostructured surface differs from the porous‐coated constructs reported in previous revision studies and is more cost‐effective. Implant survivorship for re‐revision was 94.1% and 90.2% at 5 and 10 years, respectively, and 99.0% and 95.0% for aseptic loosening. These findings indicate robust osseous integration and durable fixation despite the absence of a porous coating. Of note, the increase in cumulative risk near 10 years was driven by a single late re‐revision event, which may lead to underestimation of long‐term survivorship due to reduced precision at the tail of the curve.

This study provides a detailed analysis of the reoperation rate, capturing any subsequent surgical intervention irrespective of implant retention. The 10‐year cumulative incidence of reoperation was 21.7%, underscoring the clinical burden of secondary procedures and providing valuable context for patient counselling. Direct comparison with previous studies is challenging, as reoperation is not uniformly defined or reported in the revision THA literature. Most reoperations in the present cohort were related to infection or instability rather than mechanical acetabular failure. Reporting reoperations alongside implant survival, therefore, represents a strength of this study and reflects clinically relevant outcomes beyond implant failure alone.

Previous revision surgery was the only factor associated with an increased risk of subsequent failure in the present cohort, whereas demographic and surgical factors as well as revision indication showed no relevant association with outcome. Although the precision of the risk estimates is limited by the small number of events, the association was consistent across analyses, suggesting an effect of revision burden. These findings are consistent with large registry studies and meta‐analyses demonstrating a stepwise increase in re‐revision risk with each additional revision procedure [6, 26]. Clinically, this underscores that revision burden is a major determinant of implant survivorship and highlights the need for careful consideration of indications for further revision surgery and thorough patient counselling.

Functional outcome data following rTHA are limited, and comparisons across studies are challenging by heterogeneity in cohorts, indications and implant systems. Reported postoperative HHSs typically range from 70 to 90, indicating moderate to good function [4, 11, 17, 23]. The mean HHS of 76 and a median UCLA activity score of 6 observed in the present study are consistent with these findings and align with previously reported outcomes after revision THA [11, 19, 28]. These results suggest functional outcomes comparable to those reported in the literature and add to the limited evidence on patient‐reported outcomes following acetabular revision surgery.

This retrospective single‐centre study has inherent limitations and limited generalisability, including the cohort size, absence of a control group and a clinically heterogeneous revision population comprising septic and aseptic revisions, isolated acetabular and combined revisions, as well as varying reconstructive strategies and implant configurations. The relatively small number of re‐revision events limits the statistical power of the risk factor analyses and warrants cautious interpretation of the regression results. Although competing‐risk methodology represents a methodological strength by reducing overestimation of implant survivorship due to unrelated deaths, selection bias cannot be excluded, as the decision to use a primary press‐fit cup was based on preoperative imaging, intraoperative assessment, bone stock, defect morphology and surgeon judgment.

Furthermore, interpretation of the functional and radiographic outcomes is limited by restricted follow‐up availability. Preoperative PROMs were not consistently available, postoperative PROM assessment was obtained only in a subset of surviving patients at latest follow‐up, and radiographic follow‐up was likewise available only for a subset of patients, introducing the potential for survivorship bias and limiting longitudinal assessment. In addition, a formal interobserver reliability analysis for radiographic assessment was not performed. Nevertheless, this study provides one of the first mid‐ to long‐term evaluations of this specific cementless press‐fit, non–porous‐coated acetabular cup used in a revision setting.

## CONCLUSIONS

In selected acetabular revision cases with sufficient bone stock, the use of a cementless primary press‐fit acetabular cup achieved good mid‐ to long‐term implant survivorship, while postoperative functional outcomes were comparable to those reported in the existing revision THA literature. Aseptic loosening accounted for only a minority of failures, indicating reliable biological fixation in this revision setting. Within this context, the present findings suggest that primary press‐fit cups represent a reasonable alternative to revision‐specific implants in appropriately selected cases.

## AUTHOR CONTRIBUTIONS

**Kevin‐Arno Koch**: Conceptualisation; investigation; formal analysis; visualisation; writing—original draft preparation. **Benjamin Zimmer**: Investigation; data curation. **Raphael Trefzer**: Writing—review and editing. **Mustafa Hariri**: Writing—review and editing. **Tilman Walker**: Investigation; writing—review and editing. **Christian Merle**: Conceptualisation; methodology; investigation; writing—review and editing. **Johannes Weishorn**: Investigation; writing—review and editing. **David Spranz**: Conceptualisation; writing—review and editing; supervision. All authors read and approved the final manuscript.

## FUNDING INFORMATION

The authors have no funding to report.

## CONFLICTS OF INTEREST STATEMENT

Christian Merle declares the following conflicts of interest outside the submitted work: Speakers bureau/paid presentations and paid consultant for ZimmerBiomet and Medacta. Financial and material support from LINK, Heraeus and Mölnlycke. Board member/committee appointments for AE Germany. The remaining authors declare no conflict of interest.

## ETHICS STATEMENT

The current study was approved by the Ethics Commission of the Medical Centre, University of Heidelberg (S‐496/2011). Written informed consent was obtained from every patient before inclusion.

## Data Availability

The data will be available upon reasonable request.
